# Supplementary material for: Apheresis Efficacy and Tolerance in the Setting of HLA-Incompatible Kidney Transplantation
Source: J Clin Med. 2021 Mar 23;10(6):1316. doi: 10.3390/jcm10061316 (PMC8005077; doi:10.3390/jcm10061316)

Supplementary Table 1: Mixed model modeling the effect of session type as a fixed and a random effect on DSA class I reduction

	Fixed model		Mixed model		Variance ± SD
Inter-session MFI of immunodominant class I DSAs	Plasma exchange<br>Immunoadsorption<br>Volume of treated plasma	p=0.829<br>**p=0.035**<br>**p=0.047**	Plasma exchange<br>Immunoadsorption<br>Volume of treated plasma	p=0.829<br>**p=0.035**<br>**p=0.047**	1x 10-3 ± 0.03<br>3x 10-4 ± 0.01

Supplementary Table 2 Mixed model modeling the effect of session type as a fixed and a random effect on DSA class II reduction

	Fixed model		Mixed model		Variance ± SD
Inter-session MFI of immunodominant class II DSAs	Plasma exchange<br>Immunoadsorption<br>Volume of treated plasma	p=0.180<br>p=0.388<br>**p=0.022**	Plasma exchange<br>Immunoadsorption<br>Volume of treated plasma	p=0.195<br>p=0.375<br>**p=0.018**	133 ± 11<br>22 ± 5

Supplementary Figure 1 : Percentage of IgM, IgA and IgG reduction post immunoadsorption alone or combined with membrane filtration

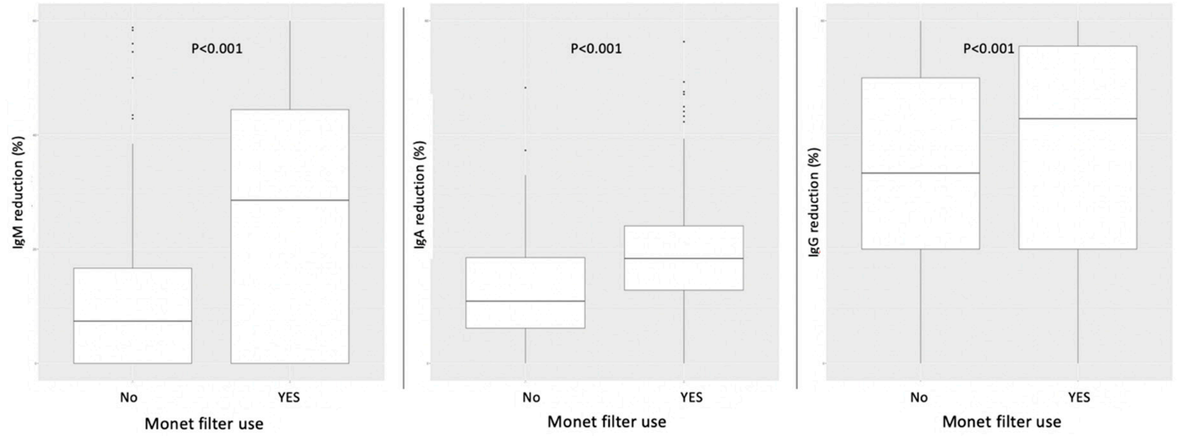

Supplement: Supplementary file 1 [file jcm-10-01316-s001.pdf]
